# Gold Nanoparticles Used as Protein Scavengers Enhance Surface Plasmon Resonance Signal

**DOI:** 10.3390/s17122765

**Published:** 2017-11-29

**Authors:** Erenildo Ferreira de Macedo, Daniela Maria Ducatti Formaggio, Nivia Salles Santos, Dayane Batista Tada

**Affiliations:** Instituto de Ciência e Tecnologia, Universidade Federal de São Paulo, Rua Talim 330, Vila Nair, São José dos Campos, SP 12231-280, Brazil; e.macedo@unifesp.br (E.F.d.M.); dformaggio@unifesp.br (D.M.D.F.); nivia.salles@unifesp.br (N.S.S.)

**Keywords:** surface plasmon resonance, gold nanoparticles, signal amplification, lectins, concanavalin A, peanut agglutinin

## Abstract

Although several researchers had reported on methodologies for surface plasmon resonance (SPR) signal amplification based on the use of nanoparticles (NPs), the majority addressed the sandwich technique and low protein concentration. In this work, a different approach for SPR signal enhancement based on the use of gold NPs was evaluated. The method was used in the detection of two lectins, peanut agglutinin (PNA) and concanavalin A (ConA). Gold NPs were functionalized with antibodies anti-PNA and anti-ConA, and these NPs were used as protein scavengers in a solution. After being incubated with solutions of PNA or ConA, the gold NPs coupled with the collected lectins were injected on the sensor containing the immobilized antibodies. The signal amplification provided by this method was compared to the signal amplification provided by the direct coupling of PNA and ConA to gold NPs. Furthermore, both methods, direct coupling and gold NPs as protein scavengers, were compared to the direct detection of PNA and ConA in solution. Compared to the analysis of free protein, the direct coupling of PNA and ConA to gold NPs resulted in a signal amplification of 10–40-fold and a 13-fold decrease of the limit of detection (LOD), whereas the use of gold NPs as protein scavengers resulted in an SPR signal 40–50-times higher and an LOD 64-times lower.

## 1. Introduction

Surface plasmon resonance is a well-established method to study qualitatively and quantitatively the interaction between biomolecules based on the measurement of small variations of the refractive index in the surrounding dielectric medium of a thin metallic film irradiated with light at a certain angle [1]. The SPR technique stands out from other analytical techniques for being a real-time and a label-free technique that allows the quantification of the kinetics and thermodynamics parameters of interactions between two molecules. In the last two decades, SPR had been very useful in life and materials sciences. Despite its wide applications in biomedical, biotechnology (proteomics, genomics), clinical diagnosis and materials engineering, much effort has been currently dedicated to the enhancement of SPR sensitivity aiming at a very low limit of detection [2,3,4,5,6,7,8,9,10].

Most of the recently-developed strategies for the enhancement of SPR sensitivity have been based on nanotechnology for designing a sensor surface with a higher surface area, as well as for employing nanoparticles (NPs) as amplification agents. These strategies have been recently reviewed by several authors [11,12,13,14,15]. The use of metallic NPs, especially gold, has been pointed out as the methodology with the highest SPR sensitivity and lowest limit of detection. The great performance of gold NPs as SPR signal amplifiers has been attributed to the localized surface plasmon coupling.

The understanding of how the localized surface plasmon (LSP) of the NP interacts with the delocalized thin film surface plasmon polariton (SPP) has stimulated a number of theoretical and experimental studies [15,16,17,18,19,20,21]. Theoretical studies have been based on Mie’s theory, which describes the enhancement of the electromagnetic field by light scattering by a sphere, which was demonstrated by solving Maxwell’s equation. The electric field of the incident light can be considered constant if the radius of the particle is considerably smaller than the wavelength of the incident light. In this case, the electrons excited by the incident light absorb the energy and oscillate under the influence of the electromagnetic field of the incident electromagnetic wave. Mock et al. [16] succeeded in probing the coupling effects by measuring the light scattered from individual gold NP placed close to the gold thin film. Their results could be related to the numerical findings achieved by Leveque et al. [17]. By combining theoretical studies with experimental assays, the plasmon coupling between metal NPs and metal thin film has been unveiled. It has been elucidated that the metal film induces a polarization to the single nanoparticle light scattering, and this effect is especially strong when the NP is within a distance of 50 nm from the film surface [16]. As a consequence, highly sensitive wavelength shifts and polarization effects in the scattered light are observed after the incidence of light on the thin film (passing through a prism as occurs in the SPR biochip). Briefly, the incident light excites SPP of the thin film. When coupled with NPs, the system enables the light to scatter into the far-field. The LSP, in turn, is also excited by the evanescent field created by the resonance between the incident light and the SPP. Thus, both resonance effects take place simultaneously. Although this mechanism of plasmon coupling has been extensively studied in the light of electromagnetic theory, as the distance between two particles or between one NP and a thin layer surface approaches the subnanometer scale, the quantum nature of the system has to be taken into account [22].

**Figure 1:**
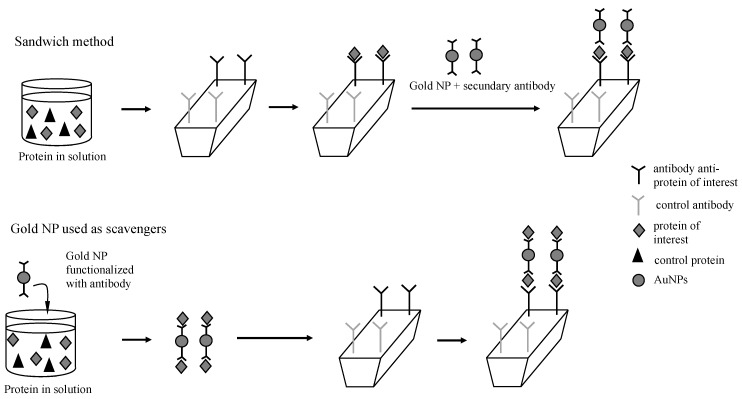
Comparative representation of the application of gold NPs in the sandwich method, where the proteins in solution are directly injected on the biosensor, and the application of gold NPs as protein scavengers, where the NPs are firstly mixed with the proteins in solution and then injected on the biosensor.

In the analysis of chemical and biochemical systems by the SPR technique, gold NPs have been applied on sensor surfaces, as well as analyte tags [5,6,7,8,10,23,24,25,26,27,28,29]. Regarding SPR sensitivity, the use of gold NPs has provided over a 103-times better sensitivity, and in terms of limit of detection, it has afforded the detection of proteins in a range lower than picomolar [5,6,7,8]. The majority of the recently-reported strategies addressed the biofunctionalization of gold NPs for application in sandwich assays. For this purpose, gold NPs functionalized with a secondary antibody were able to tag the analyte after it recognized the antibody immobilized on the sensor (Figure 1).

**Figure 2:**
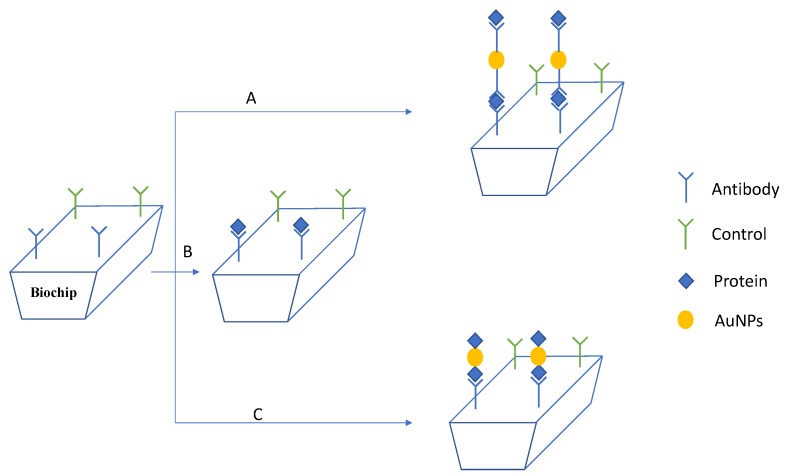
Representation of the SPR assays: Firstly, the biosensor was functionalized with antibody anti-ConA and anti-PNA. Following, (**A**) protein captured by AuNPs functionalized with antibody anti-ConA or anti-PNA, (**B**) protein in solution or (**C**) protein covalently linked to AuNPs was injected on the biosensor.

Despite the broad utility and the great performance, sandwich assays are indirect measurements that require several experimental steps and reactants. Herein, we compared two simpler methods of SPR signal amplification by using gold NPs (Figure 2). Two sets of lectin-antibody were used to evaluate the methodologies: peanut agglutinin (PNA)-antibody anti-PNA and concanavalin A (ConA)-antibody anti-ConA. Due to the expanded range of applications of lectins in the life sciences, biosensors for lectins’ detection have been the focus of several research works [30,31,32]. The first method studied here was the covalent linking of PNA and ConA on gold NPs before the proteins were injected on the SPR system (Figure 2). Even though this method has the advantage of being the simplest one, it would be limited to purified protein solutions. When the analyte is in a solution containing other proteins or biomolecules, as for example in the biological serum, this method would be unable to distinguish the protein of interest from the others. In these cases, the method employing gold NPs as protein scavengers in a solution of multiple components would be useful for collecting the protein of interest, as well as for enhancing the SPR signal (Figure 1). In this method, the gold NPs were functionalized with the antibody correspondent to the protein of interest, and after being incubated with the sample, the gold NPs could selectively capture the analyte. Then, gold NPs coupled with the protein would be detected by the antibody immobilized on the sensor (Figure 2). Both methods were compared to each other, as well as to the analysis of free protein in solution regarding the variation of reflectivity and the limit of detection.

The methods of signal enhancement based on gold NPs were evaluated for protein detection at a low concentration (nanomolar), as well as at a high protein concentration (micromolar). Even if the contribution of signal amplification is most important in the analysis of samples containing proteins of low molecular weight or proteins at very low concentration, the evaluation of these methods with samples at high concentration is important to define the saturation of each method. Since the strategies to enhance SPR signals employing NPs increases the mass density and the thickness of the label on the sensor, it is expected that SPR sensitivity would decrease at a certain analyte concentration due to the decrease of the evanescent wave with the increasing distance from the sensor surface.

In this work, for the first time, gold NPs were proven to be useful as protein scavengers in a sample of proteins in solution. In addition to the amplification of the SPR signal due to the localized plasmon surface coupling, the performance of this method was enriched by NPs’ ability to concentrate the protein of interest on the sensor surface.

## 2. Materials and Methods

### 2.1. Materials

All the reactants, lectins PNA and ConA and the antibodies anti-PNA and anti-ConA, were purchased from Sigma-Aldrich (São Paulo, SP, Brazil) and used as received without further purification. The solutions of proteins were prepared according to the recommendation from Sigma-Aldrich.

### 2.2. Gold NPs’ Synthesis

Gold NPs were synthesized by the Turkevich method [33]. A solution of tetrachloroauric acid (20 mL; $5\times 10^{-2}$ mol·L${}^{-1}$) was added to 1 L of bi-distilled water (Milli-Q, São Paulo, SP, Brazil) at 95 °C. Under stirring, 10 mL of aqueous solution of sodium citrate (0.3 mol·L${}^{-1}$) were added. The solution was kept at 95 °C during 15 min when it turned from yellow to red indicating the formation of gold colloid. The NP suspension was cooled in an ice-bath to 23 °C and stored at 4 °C.

### 2.3. Gold NPs’ Functionalization

Gold NPs were functionalized with lectins (PNA or ConA) and with antibodies anti-PNA and anti-ConA by using the 1-ethyl-3-(3-dimethylaminopropyl) carbodiimide hydrochloride/*N*-Hydroxysuccinimide (EDC/NHS) coupling reaction. An ethanolic solution of mercaptoundecanoic acid (MUA) was added to 990 μL of gold NP suspension. After stirring in an ultrasonic bath at room temperature for 30 min and resting of 3 h, the NP suspension was centrifuged (21,130× *g*; 30 min) and resuspended in HEPES buffer solution (10 mM; pH 7.4). Following, 10 μL of an aqueous solution of EDC (7.5 mM) and NHS (1.5 mM) were added to the NP suspension and kept at rest for 10 min. Finally, the protein to be linked to the NPs’ surface was added at the desired concentration.

### 2.4. Gold NPs’ Characterization

Gold NPs’ size and ζ-potential were measured by dynamic light scattering (Delsa Nano, Beckman Coulter, São Paulo, SP, Brazil) in aqueous suspension. The size was also calculated by analyzing gold NPs’ UV-Vis spectra (Thermo Scientific Evolution 200, São Paulo, SP, Brazil) according to Haiss et al. [34].

### 2.5. Sensor Preparation

Before use, all the SPR chip sensors (Horiba SPRi-Biochip, Paris, France) were cleaned with piranha solution composed of 15 mL concentrated H${}_{2}$SO${}_{4}$ and 5 mL of H${}_{2}$O${}_{2}$. After exhaustively washing with deionized water and ethanol, the sensor was immersed overnight in an ethanolic solution of MUA (1.6 mM) and 6-mercaptohexanol (8 mM). Following that, the sensor was washed with ethanol and dried under Ar stream. In order to immobilize the antibodies, the sensor was immersed in an aqueous solution of EDC (60 mM) and NHS (22 mM) for 30 min. The sensor was then washed with deionized water and dried under Ar stream. The antibodies were immobilized on small spots on the sensor surface by dropping the proteins solution (100 μg·mL${}^{-1}$) with a 10-μL micropipette. Finally, the sensor was kept at rest during 12 h inside Petri dishes filled with 2 mL of water; a volume enough to immerse only the basis of the sensor and keep the humidity. After washing the sensor with HEPES buffer solution, it was connected to the SPR system (Horiba SPRi Lab+, Paris, France).

### 2.6. SPR Assays

The SPR assays were performed in the Horiba SPRi Lab+ equipment where the SPR effect was obtained under the irradiation of the biochip by a 635-nm light-emitting diode. HEPES buffer solution at 10 mM (pH 7.4) was used as the running buffer, and a solution of bovine serum albumin (BSA) 1% in the same buffer solution was used to block the naked areas on sensor surface. After the injections of 200 μL of proteins in solution (35 μL/min), the sensor was regenerated by injecting an aqueous solution of glycine (0.1 M; pH 2.0). The same protocol was followed in the assays with gold NPs functionalized with PNA or ConA. In the assays with gold NPs applied as protein scavengers, before the NPs were injected into the system, they were incubated overnight at room temperature with the solution of proteins (Figure 2). After being injected into the system, the sensor was regenerated with glycine (0.1 M; pH 2.0). All the SPR data were analyzed by using Origin 6.0 and MATLAB 2015 software. The values of the variation of reflectivity were calculated by using the data of the kinetic curves after the injection of buffer solution and the stabilization of the system. The buffer solution was used as the washing solution in order to washout the molecules or NPs weakly bound to the biochip. If the molecules or NPs were washed out by the buffer solution, it is probable that they were only adsorbed on the surface. In this way, the gold NPs that adsorbed on the sensor, but were not interacting with the proteins linked to the sensor were washed out by the buffer solution, and the values presented in the bars corresponded to the interaction of molecules or NPs with the proteins on the biochip.

### 2.7. Calculation of the Limit of Detection

The limit of detection of each method was calculated after performing SPR assays with solutions of PNA, PNA covalently linked to gold NPs and PNA captured by gold NPs at the concentrations of 95 nM, 142.5 nM, 190 nM, 285 nM and 380 nM. The average value of variation of reflectivity and the standard deviation of each measurement were used to construct the analytical curve as a function of protein concentration. Linear fitting was applied at the linear range of the analytical curve, and the slope was used in Equation (1). The SPR assays were also performed with blank samples that were composed of solutions without protein or gold NP suspension incubated with solutions without protein. The standard deviation of the values of variation of reflectivity observed in those assays was used in Equation (1) in order to calculate LOD values for each method.

(1) $$LOD=\frac{3\times Sblank}{B}$$

where *Sblank* is the standard deviation of the measures with blank samples and *B* is the slope of linear fitting of the variation of reflectivity vs. protein concentration graph. In order to calculate the LOD of the method based on the localized surface plasmon resonance (LSPR) shift, gold NPs functionalized with antibody anti-PNA were incubated with PNA solutions at 0.00 nM, 23.75 nM, 47.50 nM, 95.00 nM and 190.00 nM. After the incubation, the NPs were centrifuged (21,130× *g*; 30 min), the supernatant was withdrawn and the NPs were resuspended in HEPES buffer. Then, UV-Vis spectra of gold NPs were obtained and the value of λmax of functionalized gold NP was subtracted from the value of λmax measured for the gold NPs incubated with PNA solution resulting in the value of LSPR shift. The measurements were done in triplicate. The value of LSPR shift at 0.00 nM of PNA was used as a blank. A graph of LSPR shift as a function of PNA concentration was constructed, and after the linear fitting, the value of LOD was calculated according to Equation (1), the same used to calculate the LOD of the SPR.

## 3. Results

### 3.1. Characterization of Gold NPs

**Figure 3:**
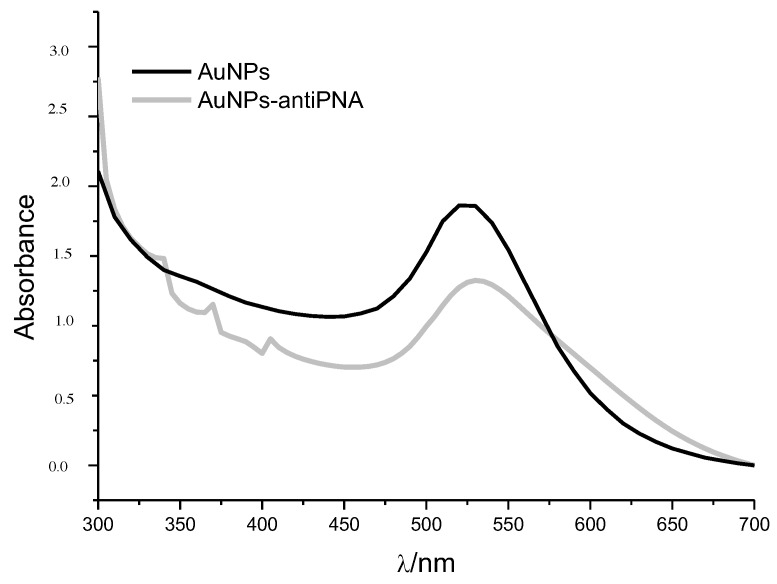
UV-Vis spectrum of gold NPs before (AuNPs) and after the functionalization with antibody anti-PNA (AuNPs-antiPNA).

The mean hydrodynamic diameter of gold NPs measured by DLS was (23 ± 8) nm, which was in line with the mean diameter calculated by analyzing NPs’ UV-Vis spectrum. According to Haiss et al. [34], the λmax identified at 520 nm was characteristic of 20 nm-diameter NPs (Figure 3). The narrow plasmon absorbance band observed in the UV-Vis spectrum and the low polydispersity index calculated by DLS (PDI = 0.21) indicated the absence of NP aggregates and the homogeneous distribution of the size of gold NPs in suspension. The value of the ζ-potential measured by DLS was −36 mV, indicating a negatively-charged surface, which was already expected since the NPs are stabilized by citrate molecules. The high ζ-potential provided satisfactory stability of the NP suspension. A 10-nm red-shift of the λmax (530 nm) was observed after gold NP functionalization with the proteins PNA or ConA and the antibodies anti-PNA or anti-ConA (Figure 3). Red-shift of the SPR absorption peak is a well-known effect of the increasing of particle size or binding of molecules to gold NPs’ surface, and for this reason, it could be considered as indicative that the functionalization process was successful. Another indication that the proteins were linked to the gold NPs was the increase in the hydrodynamic diameter; the size measured by DLS was 60 nm. No significant change was observed in the ζ-potential, measured as −30 mV, which could explain the fact that the NP suspension continued to be stable after the functionalization.

### 3.2. SPR Signal Amplification by Gold NPs

**Figure 4:**
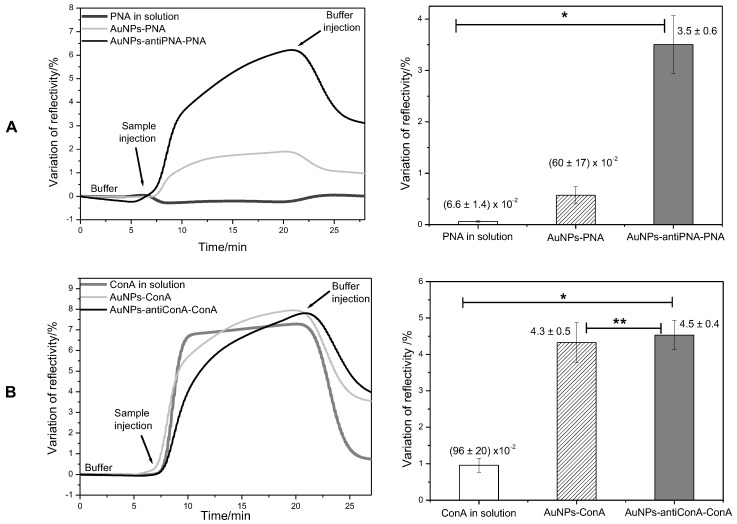
Kinetics curves and mean value of the variation of reflectivity on spots of antibody anti-PNA (**A**) and on spots of antibody anti-ConA (**B**) after the injection of proteins in solution (dark gray), proteins covalently linked to gold NPs (light gray) and proteins captured by gold NPs functionalized with antibody anti-PNA or anti-ConA (black) at the concentration of 94 nM. * Values significantly different (ANOVA; p<0.05). ** Values not significantly different (ANOVA; p<0.05).

The signal amplification provided by the gold NPs covalently linked to the proteins was evaluated by comparing the SPR signal of the direct assay of protein in solution to the signal generated in the assay with proteins linked to the gold NPs. Firstly, the SPR response was analyzed for the detection of 95 nM of PNA and ConA. Figure 4 shows the kinetic curves of the interaction of PNA and ConA with the antibody anti-PNA and anti-ConA. In both cases, it was possible to observe the interaction of the lectins with the respective antibodies. However, the signal generated in the assay with lectins in solution was very weak and noisy, indicating that even if the proteins could be detected, the low signal/noise ratio could compromise the accuracy of the analysis. Nevertheless, when the proteins were injected, linked to the gold NPs, the noise was negligible, and in comparison to the analysis of proteins in solution, the variation of reflectivity in the analysis of proteins linked to the gold NPs was 9-times and 4.3-times higher than in the detection of PNA and ConA, respectively (Figure 4).

Although the potential of gold NPs as amplifier agents when linked to the protein had been proven, this method would be limited to the cases where the protein to be analyzed would be present in a purified solution. For this reason, a second method was proposed. In the second method, gold NPs were previously functionalized with an antibody able to selectively bind the protein of interest. Although it required an additional experimental step, this method has the advantage of being useful in the analysis of a protein in a solution of mixed components, by acting as a specific protein scavenger. This method was evaluated in assays for the detection of PNA and ConA. When gold NPs functionalized with antibody anti-PNA were incubated with a solution of PNA 95 nM and then injected in the SPR system, the variation of reflectivity was of 3.5%, a value 53-times higher than the variation observed after the injection of protein in solution (ΔR = $6.6\times 10^{-2}$%) and about six-times higher than the variation of reflectivity observed after the injection of PNA covalently linked to the gold NPs (ΔR = $60\times 10^{-2}$%) (Figure 4A).

In the assay of ConA, the use of gold NPs functionalized with antibody anti-ConA provided a signal amplification of 4.7-times compared to the analysis of free protein in solution. This amplification was very similar to that observed when the protein was covalently linked to the gold NPs (Figure 4B).

The amplification of the SPR signal can be attributed to the coupling between the localized surface plasmon resonance of gold NPs and the gold surface of the biosensor. Additionally, when the protein linked to the NPs interacts with the biomolecules on the sensor, the variation of the refractive index is higher due to the increased coverage area compared to the interaction of free protein. The use of gold NPs functionalized with antibodies as protein scavengers has a further factor that enhances the SPR signal, that is the concentration of protein on the NPs surface. When a dilute solution of protein is directly analyzed in the SPR system, the signal can be very weak or even absent. However, when the bio-functionalized gold NPs were previously incubated with the solution of proteins before the analysis, the concentration of protein injected in the SPR system was increased, and its interaction with the sensor enhanced the binding mass; consequently, the variation of the refractive index was higher.

Despite the innumerable works addressing the use of gold NPs as the SPR signal amplification agent, most them used the sandwich method at a very low concentration of the analyte. However, it is known that the SPR sensibility decays with the distance from the sensor surface due to the decrease of the evanescent wave. For this reason, the methods are very useful when the analyte is at a very low concentration, but when this concentration is not known and the protein of interest may be at high concentration, its quantification would probably be underestimated. Therefore, it is very important that the methods for signal amplification be evaluated at higher concentrations of protein.

**Figure 5:**
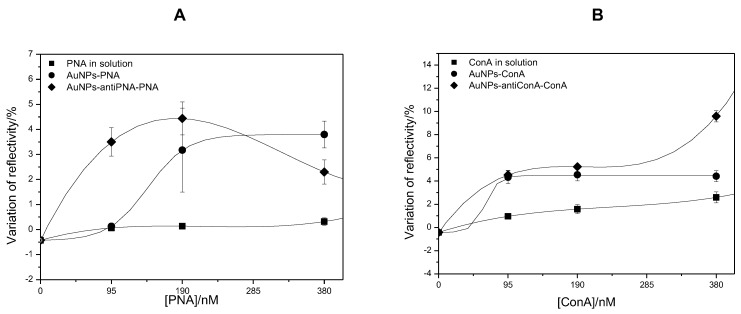
Mean value and standard deviation of the variation of reflectivity on spots of antibody anti-PNA (**A**) and antibody anti-ConA (**B**) after injection of free PNA (**A**) and ConA (**B**) in solution, covalently linked to gold NPs and captured by gold NPs functionalized with anti-PNA or anti-ConA.

In this work, both methods, protein linked to gold NPs and gold NPs used as protein scavengers, were tested with solutions of increasing protein concentrations. Besides the solutions at 95 nM of PNA and ConA, solutions of 190 and 380 nM were also analyzed. Figure 5 shows the mean values of the variation of reflectivity as a function of the concentration of PNA (Figure 5A) and ConA (Figure 5B). It was observed that the sensitivity was compromised for protein concentrations higher than 190 nM, although in the analysis of ConA at 380 nM, the use of gold NPs as protein scavengers had been able to provide an SPR signal of 9.6%, a value 8-times higher than the signal observed in the analysis of the free ConA in solution (ΔR = 2.5%) at the same concentration. The absence of linearity at the concentration range of 95–380 nM showed that both methods based on the signal amplification by gold NPs could be useful in the analytical quantification of PNA and ConA at a low concentration, but not at a higher concentration. Nevertheless, both methods were proven to be very useful in the detection of proteins at low and high concentrations since the SPR signal afforded by these methods was more than 10-times higher than the signal observed in the detection of protein in solution.

### 3.3. Limit of Detection

**Figure 6:**
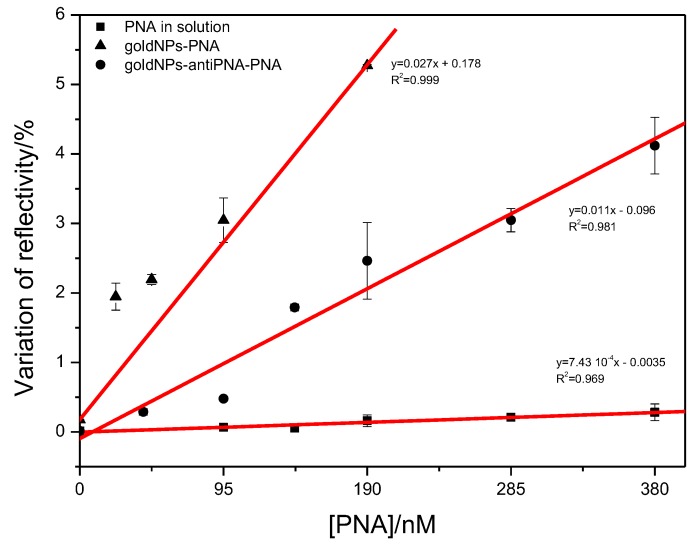
Average values and standard deviation of the variation of reflectivity as a function of the concentration of PNA detected by SPR. Linear fitting was applied for each method (red line). The linear equation and the correlation coefficient calculated for each method are displayed beside the correspondent data.

The limit of detection (LOD) is defined as the concentration of analyte that produces sensor output corresponding to three standard deviations of sensor output measured for a blank sample [1]. LOD is one of the most important parameters used to evaluate the performance of a method applied in the SPR technique. Therefore, the methods studied here were also compared regarding the LOD. SPR assays were performed with a solution of PNA at a concentration lower than 95 nM. At this range of concentration, the analytical curves could be fitted to linear equation, and the values of LOD were calculated by applying Equation (1). In the detection of free protein and protein linked to the gold NPs, the linear range of the analytical curve was extended to a higher concentration, and the linear fit was done for the analysis of 0–380 nM of PNA (Figure 6). The value of LOD calculated for the detection of free protein in solution was 45 nM, a value very similar to the values reported before for the detection of proteins by SPR [1]. As already expected, due to the enhancement of the SPR signal by gold NPs, the LOD decreased for the detection of PNA linked to the NPs. The LOD was calculated as 3.5 nM when the PNA was directly coupled to the gold NP, and an even lower LOD was observed in the detection of PNA captured by gold NPs. In this case, the LOD was calculated as 0.7 nM, a value 64-times lower than the value found for the free protein and five-times lower than the detection of PNA linked to gold NPs. These results showed that, in addition to the advantage of detecting protein in a solution of multiple components, the use of gold NPs as protein scavengers allows the detection of proteins with very low LOD.

**Figure 7:**
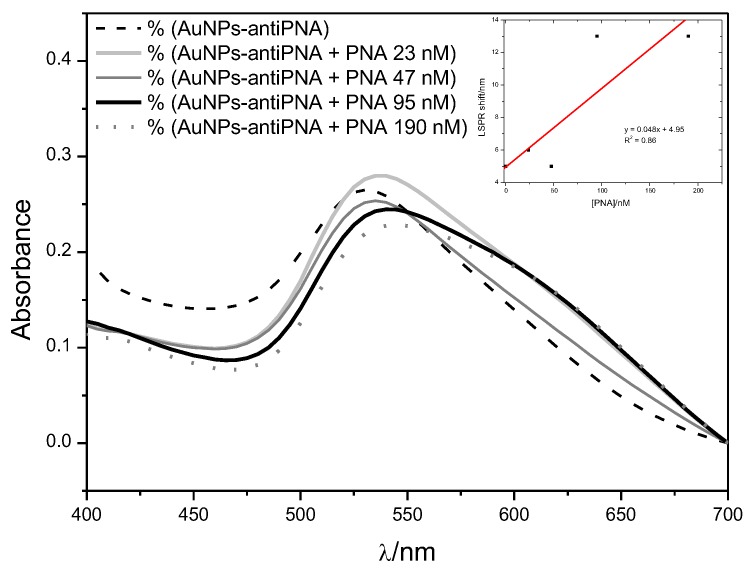
UV-Vis spectra of gold NPs functionalized with antibody anti-PNA after incubation with PNA solution at different concentrations. Insert: Linear fitting of LSPR shift of gold NPs functionalized with antibody anti-PNA as a function of the concentration of PNA in solution.

Since the LSPR shift of gold NPs has been used as an analytical method to quantify chemical and biochemical compounds in solution [35,36,37,38,39], we measured the LOD of this method in order to compare with the LOD value obtained in SPR assays. The UV-Vis spectra of gold NPs used as PNA scavenger in solution showed a red-shift of LSPR as a function of PNA concentration. However, the data presented a poor fitting to the linear equation (Figure 7), but even so, they were used to calculate the LOD by Equation (1). We have found an LOD of 500 nM for the method based on the monitoring of the LSPR shift. Notably, this value was 50-times higher than the LOD value reported by Chen et al. [40] (10 nM) for the detection of prostate-specific antigen (PSA). However, differently from the UV-Vis assay reported here (20-nm gold NPs in solution), the authors used 2.2-nm gold NPs immobilized on a prism surface. Lower values of LOD could be achieved by using NPs of different morphologies. Jana et al. [41] achieved an LOD of 10${}^{-18}$ M in the detection of PSA in serum by using gold nanostars as amplifying agents. Rodriguez-Lorenzo et al. [42] achieved an even lower LOD ($4\times 10^{-20}$ M) by using gold nanostar and silver nanocrystals as capping agents in a sandwich assay. Although lower LOD have been reported by using different setups for the detection of protein based on LSPR shifting, the setup used here had the purpose of providing a comparison between the LSPR shifting and the use of gold NPs as protein scavengers in the SPR technique. Therefore, gold NPs were used in solution in order to capture protein in a multi-component solution. The LOD of 500 nM achieved by the measurement of LSPR shift by UV-Vis was more than 500-times higher than the value of LOD of the SPR assay by applying gold NPs as protein scavengers. This result suggested that for the detection of PNA at the studied concentration range, SPR would be more appropriate than the monitoring of LSPR shift by UV-Vis spectroscopy.

### 3.4. Sensor Selectivity

**Figure 8:**
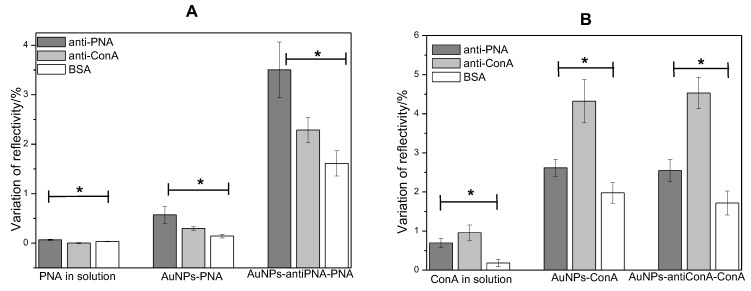
Mean values and standard deviation of the variation of reflectivity on spots of antibody anti-PNA (**A**) and antibody anti-ConA (**B**) after the injection of PNA (**A**) and ConA (**B**) at the concentration of 95 nM in solution, covalently linked to gold NPs and captured by gold NPs functionalized with anti-PNA or anti-ConA. * Values significantly different (ANOVA, p<0.05).

In order to evaluate the selectivity of the biosensor system based on the use of gold NPs as amplifier agents, the interaction of the lectins with the control proteins was compared to the interaction between the lectins and their respective antibodies. Antibody anti-ConA was used as the negative control in the assays with PNA, and the antibody anti-PNA was used as the negative control in the assays with ConA. The protein BSA was used to block the naked area of the gold surface area on the biosensor, and the interaction of the analytes on this area was also analyzed. Figure 8 shows the average value of the variation of reflectivity on the spots of antibody anti-PNA, anti-ConA and BSA after the injection of the solution of lectins, gold NPs functionalized with PNA or ConA and gold NPs used as lectin scavengers. It was observed that the reflectivity only changed a little on the BSA spots, indicating that the adsorption of PNA or ConA on these areas was very low when the lectins were in solution or conjugated with gold NPs. The highest value of variation of reflectivity on BSA spots was observed in the analysis of PNA in solution, which was two-times lower than the variation of reflectivity observed on anti-PNA spots. The lowest interaction with BSA was observed in the ConA assay, where the variation of reflectivity was 5.3-times higher on anti-ConA spots than on BSA spots. Thus, BSA was efficient in blocking the surface and in avoiding unspecific adsorption of the lectins on the sensor.

The comparison of the interaction with the specific antibody to the interaction with the antibody used as the negative control revealed that the lectins interacted preferably with their respective antibody when the proteins were in solution or conjugated with gold NPs. However, the variation of reflectivity on the spots of the antibodies used as a negative control was higher than the variation observed on BSA spots. The high similarity among the molecular structures of PNA and ConA makes their respective antibodies able to recognize both proteins, but with different affinity and extension. Nevertheless, the SPR signals observed on the spots of antibodies anti-PNA and anti-ConA after the injection of respective lectins were about 30–50% higher than the signals observed on the spots of the negative control (Figure 8).

These results regarding the sensor specificity showed that the use of gold NPs did not increase the unspecific interactions of the proteins, and both methods could distinguish PNA from ConA. As a conclusion, in addition to the signal amplification, the methods based on the use of gold NPs presented a satisfactory selectivity.

## 4. Discussion

Recent works have reported on technological approaches for improving SPR sensitivity. Most of these works have focused on the use of the sandwich assay employing gold NPs as the signal amplifier agents [1,6,7,8,15,23,38]. It has been shown that the amplification depends on the size, composition and morphology of the NPs, as well as on the molecular weight of the protein to be detected. Although the methods had been successful in lowering the limit of detection from the micromolar to the picomolar range, these methods required many experimental steps like the labeling of the analyte or of the probe, as well as the NPs functionalization with a secondary antibody. In addition to the disadvantages brought by an indirect measurement such as the sandwich assay, these techniques were time-consuming and material-demanding since each experimental step may represent the loss of a significant amount of consumables. Compared to these methods, the covalent linking of a protein to the gold NPs would be much simpler, since it is a one-step process. In this work, we have shown that this method provides a 10-times amplification of the SPR signal in the detection of a protein at a 95 nM concentration. In a similar study, Lyon et al. [5] have reported a 15-times increase in SPR sensitivity by using electrostatically-bound conjugate between h-IgG and 10 nm-diameter colloidal gold. However, besides the simplicity and satisfactory signal amplification of this method, it has to be pointed out that it could be useful only in the analysis of a purified protein solution.

The application of gold NPs as protein scavengers would be useful in order to detect a protein in a sample with unknown composition. In this method, the NPs were functionalized with the antibody able to collect the protein of interest in a multi-component solution. Once incubated with the sample, these NPs are expected to couple with the analyte, and after being injected into the SPR system, it would be recognized by the antibody immobilized on the sensor. This method led to a 50-times amplification of the SPR signal compared to the analysis of the protein in solution. This amplification can be assigned not only to the surface plasmon coupling of gold NPs, but also to the ability of the NPs to concentrate the protein on their surface. A similar method had been proposed by Lyon et al. [5], who functionalized gold NPs with a secondary antibody and injected these NPs on the sensor subsequently to the injection of free h-IgG in solution. The protein, previously probed by the antibody anti-IgG immobilized on the sensor, interacted with the secondary antibody conjugated to the gold NPs. In comparison to the analysis of free protein, they found a 25-fold enhancement of SPR sensitivity in the detection of 17 nM of protein. Mitchell et al. [23] used 25-nm gold NPs functionalized with secondary antibody in order to improve the SPR signal in testosterone detection. The authors found an LOD of 15.4 pg·mL${}^{-1}$ and an enhancement of signal sensitivity of 12.5-fold compared to the detection by primary antibody alone. The higher sensitivity found in the present work (50-times enhanced) is due to the increase of the protein concentration on the gold NPs’ surface before the injection into the SPR system, which consequently increased the binding mass on the sensor. Aiming at the detection of peanut allergens by SPR, Lai et al. [43] and Mohammed et al. [44] have reported on an immunoassay that provided an LOD of 50 ng·mL${}^{-1}$ and 700 ng·mL${}^{-1}$. A similar value of LOD (0.39 μg·mL${}^{-1}$) was achieved by Huang et al. [8] in the detection of concanavalin A by a sandwich assay with graphene oxide and dextran-coated gold NPs. Herein, the application of gold NPs as protein scavengers resulted in a lower value of LOD (0.2 ng·mL${}^{-1}$) in the detection of PNA.

Despite the most evident utility of gold NPs’ enhancement of the SPR signal at a low concentration of protein, we should consider that the concentration range of a sample is evidently unknown before the analysis, and the performance of the NP-based methodologies should be also investigated at higher protein concentrations. In the present work, it was shown that both methods, protein directly linked to the NP and gold NPs used as protein scavengers, can be saturated, and there is an upper-limit protein concentration for the protein quantification. At higher protein concentrations (above 190 nM), there was no linearity in the analytical curve of the SPR technique. Concerning samples with a high concentration of analyte, if the gold NP-based methodologies are applied with the aim of quantification, it could result in underestimated protein quantification. However, the importance of the methodologies regarding the detection of the protein in a sample was clearly demonstrated.

## 5. Conclusions

Gold NPs have been demonstrated as SPR signal amplifiers by coating sensor surfaces, as well as by tagging the analytes. The majority of the gold NP-based methodologies addressed the sandwich technique, which required several experimental steps and provided an indirect measurement. In this work, we compared two simpler methods of SPR signal amplification by using gold NPs. The covalent binding of two lectins, PNA and ConA, on gold NPs resulted in variation of reflectivity of 9- and 4.5-times higher, respectively, than the signals obtained in the assays with lectins in solution. Despite its simplicity, this method would be limited to purified protein solutions. When the analyte is in a solution of other proteins or biomolecules, for example biological serum, this method would be unable to distinguish the proteins. For cases like this, the use of gold NPs as protein scavengers would be most appropriate. This method was based on the functionalization of gold NPs with the antibody correspondent to the protein of interest. The resultant NPs could selectively couple the protein of interest in a solution of multiple components. The detection of PNA and ConA through this method provided a 53-fold enhancement of the SPR signal and a 64-fold decrease of LOD compared to the analysis of these proteins in solution. The satisfactory signal amplification afforded by this methodology showed that this simpler approach of signal amplification by gold NPs could be useful in protein analysis by SPR.
